# Beta-Amyloid (Aβ_1-42_) Increases the Expression of NKCC1 in the Mouse Hippocampus

**DOI:** 10.3390/molecules27082440

**Published:** 2022-04-10

**Authors:** Patricia Lam, Chitra Vinnakota, Beatriz Calvo-Flores Guzmán, Julia Newland, Katie Peppercorn, Warren P. Tate, Henry J. Waldvogel, Richard L. M. Faull, Andrea Kwakowsky

**Affiliations:** 1 Centre for Brain Research, Department of Anatomy and Medical Imaging, Faculty of Medical and Health Sciences, University of Auckland, Auckland 1023, New Zealand; patty.lam@auckland.ac.nz (P.L.); c.vinnakota@auckland.ac.nz (C.V.); b.guzman@auckland.ac.nz (B.C.-F.G.); julia.newland@auckland.ac.nz (J.N.); h.waldvogel@auckland.ac.nz (H.J.W.); rlm.faull@auckland.ac.nz (R.L.M.F.); 2 Department of Biochemistry, University of Otago, Dunedin 9054, New Zealand; katie.peppercorn@otago.ac.nz (K.P.); warren.tate@otago.ac.nz (W.P.T.); 3 Pharmacology and Therapeutics, Galway Neuroscience Centre, School of Medicine, National University of Ireland Galway, H91 W5P7 Galway, Ireland

**Keywords:** Alzheimer’s disease, GABA, beta amyloid, KCC2, NKCC1, bumetanide

## Abstract

Alzheimer’s disease (AD) is a neurodegenerative disorder with an increasing need for developing disease-modifying treatments as current therapies only provide marginal symptomatic relief. Recent evidence suggests the γ-aminobutyric acid (GABA) neurotransmitter system undergoes remodeling in AD, disrupting the excitatory/inhibitory (E/I) balance in the brain. Altered expression levels of K-Cl-2 (KCC2) and N-K-Cl-1 (NKCC1), which are cation–chloride cotransporters (CCCs), have been implicated in disrupting GABAergic activity by regulating GABA_A_ receptor signaling polarity in several neurological disorders, but these have not yet been explored in AD. NKCC1 and KCC2 regulate intracellular chloride [Cl^−^]_i_ by accumulating and extruding Cl^−^, respectively. Increased NKCC1 expression in mature neurons has been reported in these disease conditions, and bumetanide, an NKCC1 inhibitor, is suggested to show potential therapeutic benefits. This study used primary mouse hippocampal neurons to explore if KCC2 and NKCC1 expression levels are altered following beta-amyloid (Aβ_1-42_) treatment and the potential neuroprotective effects of bumetanide. KCC2 and NKCC1 expression levels were also examined in 18-months-old male C57BL/6 mice following bilateral hippocampal Aβ_1-42_ stereotaxic injection. No change in KCC2 and NKCC1 expression levels were observed in mouse hippocampal neurons treated with 1 nM Aβ_1-42_, but NKCC1 expression increased 30-days post-Aβ_1-42_-injection in the CA1 region of the mouse hippocampus. Primary mouse hippocampal cultures were treated with 1 nM Aβ_1-42_ alone or with various concentrations of bumetanide (1 µM, 10 µM, 100 µM, 1 mM) to investigate the effect of the drug on cell viability. Aβ_1-42_ produced 53.1 ± 1.4% cell death after 5 days, and the addition of bumetanide did not reduce this. However, the drug at all concentrations significantly reduced cell viability, suggesting bumetanide is highly neurotoxic. In summary, these results suggest that chronic exposure to Aβ_1-42_ alters the balance of KCC2 and NKCC1 expression in a region-and layer-specific manner in mouse hippocampal tissue; therefore, this process most likely contributes to altered hippocampal E/I balance in this model. Furthermore, bumetanide induces hippocampal neurotoxicity, thus questioning its suitability for AD therapy. Further investigations are required to examine the effects of Aβ_1-42_ on KCC2 and NKCC1 expression and whether targeting CCCs might offer a therapeutic approach for AD.

## 1. Introduction

Alzheimer’s disease (AD) is a neurodegenerative disorder affecting the aging population. It is associated with the progressive loss of neurons within the hippocampus, resulting in the clinical manifestation of memory loss and cognitive impairment. The hallmarks of AD pathology are well-known as the progressive accumulation of extracellular beta-amyloid (Aβ) plaques and intracellular neurofibrillary tangles (NFTs), which severely affect the hippocampus in the disease [1,2]. The hippocampus is one of the first brain regions to exhibit neuronal loss and functional deficits, translating to clinical symptoms including memory loss [1]. Working memory requires synchronization of excitatory and inhibitory networks to function, thus disruptions in neuronal network synchrony can disrupt cognition [3,4,5]. It is widely acknowledged that a functional consequence of AD pathology is network dysfunction, as demonstrated through increased neuronal activity in hippocampal neurons [4,6,7,8,9]. Aβ_1-42_ has been implicated in inducing network instability by creating an excitatory/inhibitory (E/I) imbalance, but it is unclear what underlying mechanisms are involved. While studies have historically pointed to dysfunction in the cholinergic and glutamatergic neurotransmitter systems, it has become increasingly apparent that the GABAergic system is also affected in AD.

Recent evidence indicates altered GABAergic signaling may be a key player in exacerbating aberrant network activity in AD [2,10]. GABAergic interneurons were thought to be spared in AD, but evidence shows a reduction in GABAergic inhibition contributes to neuronal hyperactivity as opposed to enhanced glutamatergic activity [11,12]. In addition, research indicates that early in AD pathogenesis there is a selective loss of hippocampal GABAergic interneurons while pyramidal neurons are unaffected [13,14]. GABA signaling components, such as GABA receptors (GABARs) which mediate GABA responses, and GABA transporters (GATs) which terminate GABA signaling by clearing GABA from the synapse, are necessary for regulating GABA activity [2]. Region- and cell layer-specific alterations in the GABAergic system have been identified, specifically involving GATs and GABA_A_R subunits; indicating elaborate GABAergic remodeling in AD [2,10,15,16]. The functional consequences of these changes are currently unknown but speculated to affect cognition [16].

Extensive GABAergic remodeling is apparent in AD, but the cation–chloride cotransporters (CCCs) K-Cl-2 (KCC2) and N-K-Cl-1 (NKCC1) have also been implicated in disease progression by altering GABA signaling polarity in several neurological disorders [17]. CCCs, cotransport Cl^−^ across cellular membranes to regulate cell volume and modulate neuronal excitability. In the developing neuron NKCC1 is the primary Cl^−^ importer which cotransports 1 potassium (K^+^), 1 sodium (Na^+^), and 2 Cl^−^ into the neuron. KCC2, while lowly expressed in the immature neuron, becomes the predominant Cl^−^ exporter in mature neurons where it cotransports K^+^ and Cl^−^ in a 1:1 ratio [17,18]. Where NKCC1 is upregulated and KCC2 is downregulated in mature neurons, GABA can become excitatory and disrupt the E/I balance [18,19,20]. Evidence of an inhibitory-to-excitatory switch in GABA transmission has been demonstrated in Ts65Dn Down syndrome (DS) mice, which exhibit learning and memory deficits, as an upregulation of NKCC1 in the hippocampus was associated with excitatory GABA activity [21]. In such neurological disorders, application of the NKCC1 inhibitor, bumetanide, restored inhibitory GABA activity, improved behavioural deficits, and restored associative, spatial, and recognition memory [21]. A gain-of-function variant of the NKCC1 gene has also been identified in schizophrenic patients which may be implicated in affecting hippocampal neuron development [22,23]. Recently, Dargaei and colleagues demonstrated an upregulation of NKCC1 and downregulation of KCC2 was accompanied by excitatory GABA_A_R signaling in a Huntington’s disease (HD) mouse model [24]. Electrophysiology experiments confirmed a more positive reversal potential for GABA (E_GABA_) in these mice, which skews GABA to become excitatory [18,24,25,26,27]. The ability of bumetanide to restore learning and memory function in HD mice is speculated to involve restoring inhibitory GABAergic function essential to hippocampal-related learning and memory [24]. While the GABAergic system has not always been acknowledged as a key player in AD pathogenesis, it is possible a similar mechanism of GABAergic dysfunction occurs in AD. The expression levels of KCC2 and NKCC1 have been extensively characterized in the rat brain using in situ hybridization but have yet to be characterized in the human AD hippocampus [28,29,30,31,32,33,34,35,36,37]. The FDA-approved loop diuretic, bumetanide has potential to perform well in clinical trials and might improve learning and memory deficits in AD, but more work is required to establish its potential and understand the mechanism of action. The potential therapeutic benefit of bumetanide has not been investigated in AD, but a recent study suggests that bumetanide exposure was associated with a significantly lower AD prevalence in individuals over the age of 65 years in two independent clinical cohorts, suggesting the effectiveness of bumetanide in preventing AD [38].

This study investigated the hippocampal expression of KCC2 and NKCC1 and the potential neuroprotective properties of bumetanide in an in vitro and in vivo AD mouse model. Here we demonstrate KCC2 and NKCC1 expression is unchanged in the presence of Aβ_1-42_ following 5-day exposure in vitro, but after 30 days in vivo significantly increased NKCC1 expression. Furthermore, bumetanide does not improve the survival of primary mouse hippocampal neurons treated with neurotoxic Aβ_1-42_, but further reduces cell viability and has neurotoxic properties on healthy neurons.

## 2. Results

### 2.1. KCC2 Expression in Hippocampal Cultures

**Figure 1:**
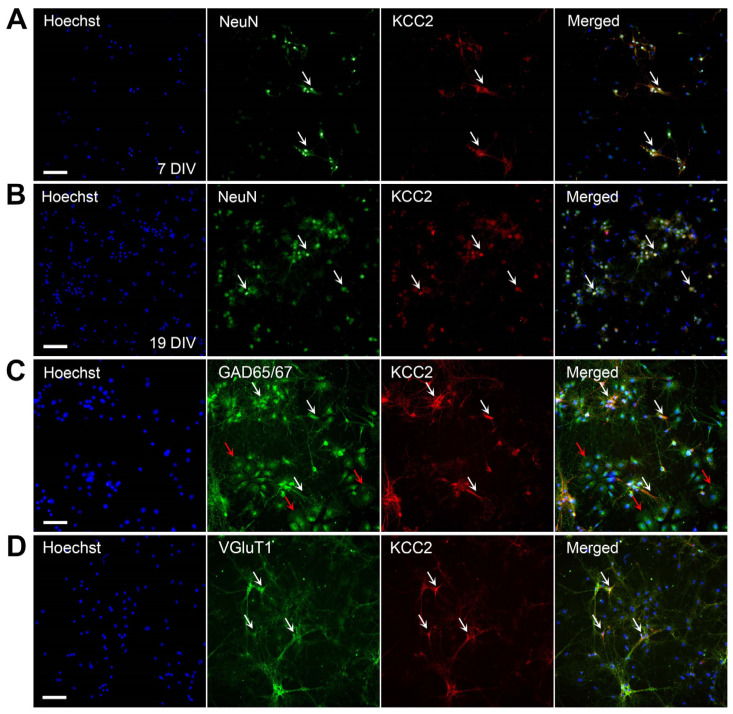
KCC2 is expressed by primary hippocampal neurons. (**A**) Neurons immunolabeled at 7 DIV; (**B**) Neurons immunolabeled at 19 DIV. Primary hippocampal neurons double-labeled for the neuronal marker, NeuN (green), and KCC2 (red) show neuronal expression of KCC2. White arrows indicate colocalization of KCC2 to neurons (yellow). (**C**) KCC2 expression in GABAergic neurons. Primary mouse hippocampal neurons at 7 DIV were double-labeled with the GABAergic marker, GAD65/67 (green), and KCC2 (red). White arrows indicate colocalization of KCC2 to GABAergic neurons (yellow). Red arrows indicate potential endothelial cells in culture which are void of KCC2 staining. (**D**) KCC2 expression in glutamatergic neurons. Primary mouse hippocampal neurons at 7 DIV were double-labeled with the glutamatergic marker, VGluT1 (green), and KCC2 (red). White arrows indicate colocalization of KCC2 to glutamatergic neurons (yellow). All cell nuclei are counterstained with Hoechst 33342 (blue). Scale bar = 100 µm.

Hippocampal cultures were fixed at 7 and 19 DIV and fluorescently labeled for NeuN and KCC2 to evaluate neuronal expression of the cotransporter. KCC2 staining was localized to the cell body with some neurons expressing KCC2 proximally and distally along neuronal processes (Figure 1A,B).

Hippocampal cultures were also double labeled with either GAD65/67 or VGluT1, and KCC2 to investigate which neuronal subtypes express KCC2. GABAergic, or GAD65/67-positive, hippocampal neurons show KCC2 expression on the cell body and sparsely along the proximal processes (Figure 1C). Based on morphology, potential endothelial cells in our cultures showed nuclear GAD65/67 expression, however, were void of KCC2 staining (Figure 1C, red arrows). Similarly, VGluT1-positive, glutamatergic neurons show KCC2 is expressed on the cell body and neuronal processes, confirming KCC2 is exclusively expressed neuronally (Figure 1D).

### 2.2. NKCC1 Expression in Hippocampal Cultures

**Figure 2:**
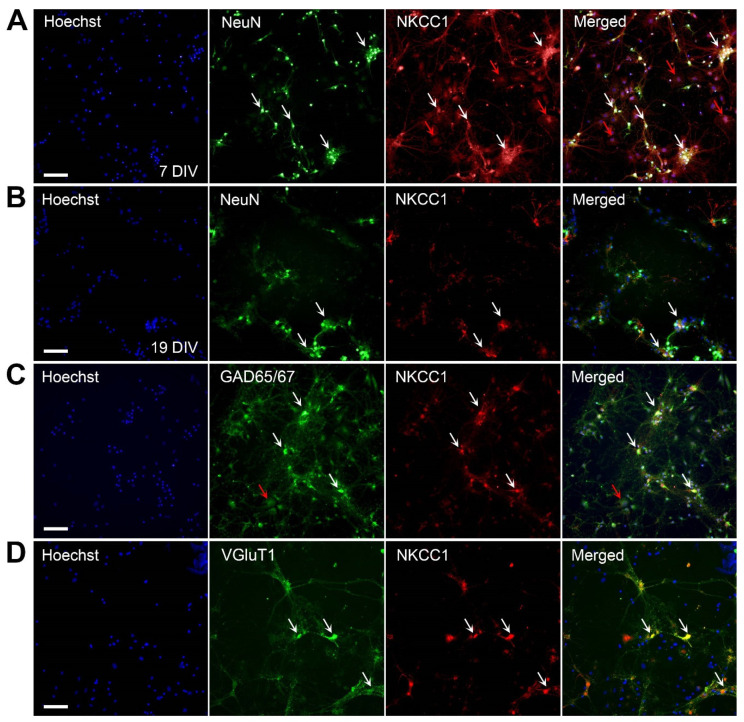
NKCC1 is expressed by primary hippocampal neurons. (**A**) Neurons labeled at 7 DIV; (**B**) Neurons labeled at 19 DIV. Primary hippocampal neurons double-labeled for the neuronal marker, NeuN (green), and NKCC1 (red) show neuronal expression of NKCC1. White arrows indicate colocalization of NKCC1 to neurons (yellow). (**C**) NKCC1 expression in GABAergic neurons. Primary mouse hippocampal neurons at 7 DIV were double-labeled with the GABAergic marker, GAD65/67 (green), and NKCC1 (red). White arrows indicate colocalization of KCC2 to GABAergic neurons (yellow). (**D**) NKCC1 expression in glutamatergic neurons. Primary mouse hippocampal neurons at 7 DIV were double-labeled with the glutamatergic marker, VGluT1 (green), and KCC2 (red). Arrows indicate colocalization of NKCC1 to glutamatergic neurons (yellow). Red arrows indicate potential endothelial cells in culture. All cell nuclei are counterstained with Hoechst 33342 (blue). Scale bar = 100 µm.

Expression of NKCC1 was also explored in hippocampal cultures by double-labeling with NeuN and NKCC1. NKCC1 staining was observed on the cell bodies and along the neuronal processes of NeuN-positive cells in these cultures (Figure 2A,B). Unlike KCC2, NKCC1 showed expression outside of NeuN-positive cells, suggesting expression on non-neuronal cells such as endothelial cells (Figure 2A,C, red arrows). At 19 DIV, the NKCC1 is weaker compared with the labeling observed at 7 DIV. Both GABAergic and glutamatergic neurons demonstrated NKCC1 expression localized to the cell body, neuronal soma, and weakly along neuronal processes (Figure 2C,D).

### 2.3. Effect of Aβ_1-42_ and Bumetanide on KCC2 and NKCC1 Expression in Hippocampal Cultures

**Figure 3:**
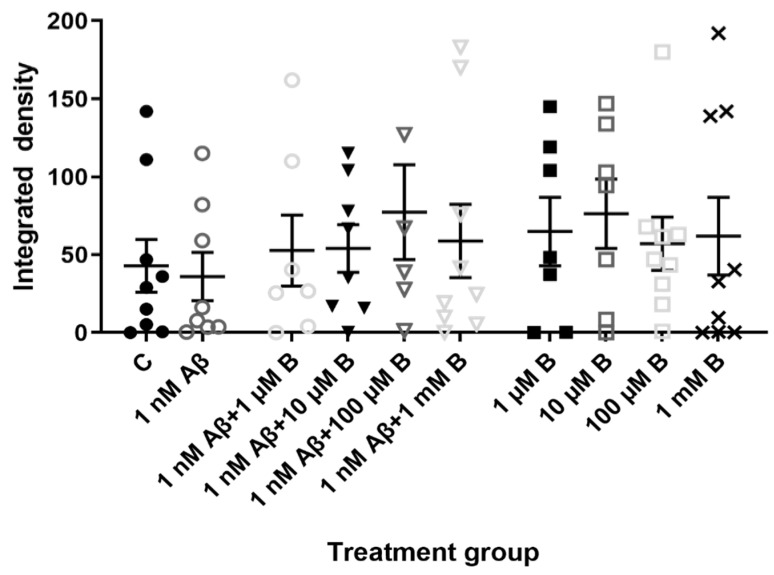
KCC2 expression in primary mouse hippocampal neurons treated with Aβ_1-42_ and bumetanide. Expression of KCC2 in primary hippocampal neurons at 19 DIV in vehicle controls (C) or following treatment with 1 nM Aβ_1-42_ (Aβ), 1 µM, 10 µM, 100 µM, and 1 mM bumetanide (B) with and without 1 nM Aβ_1-42_ for 5 days. Integrated density measurements from immunocytochemistry experiments were conducted using ImageJ Fiji software to quantify KCC2 expression on primary hippocampal neurons. Data are expressed as the mean ± SEM. One-way ANOVA with Tukey’s post hoc, *n* = 6–9 for each experimental group.

To determine the effect of Aβ_1-42_ and bumetanide on KCC2 and NKCC1 expression, hippocampal cultures treated with Aβ_1-42_ and bumetanide were triple-labeled with KCC2, NKCC1 and NeuN. Signal density measurements of KCC2 showed that 5-day treatment with 1 nM Aβ_1-42_ did not change KCC2 expression in hippocampal cultures compared to the vehicle control group (Figure 3; *p* > 0.05, *n* = 9 for controls, *n* = 8 for Aβ_1-42_). Adding bumetanide to Aβ_1-42_-treated cells at 1 µM (*p* > 0.05, *n* = 7), 10 µM (*p* > 0.05, *n* = 8), 100 µM (*p* > 0.05, *n* = 6), or 1 mM bumetanide (*p* > 0.05, *n* = 9) did not alter KCC2 expression compared to vehicle control either. Similarly, administering bumetanide alone did not alter KCC2 expression at 1 µM (*p* > 0.05, *n* = 7), 10 µM (*p* > 0.05, *n* = 7), 100 µM (*p* > 0.05, *n* = 9), or 1 mM (*p* > 0.05, *n* = 9), compared to vehicle controls. The high variability in staining density for each treatment group might indicate that subpopulations of hippocampal neurons express different levels of KCC2. Overall, KCC2 expression levels were unchanged across all treatment groups.

**Figure 4:**
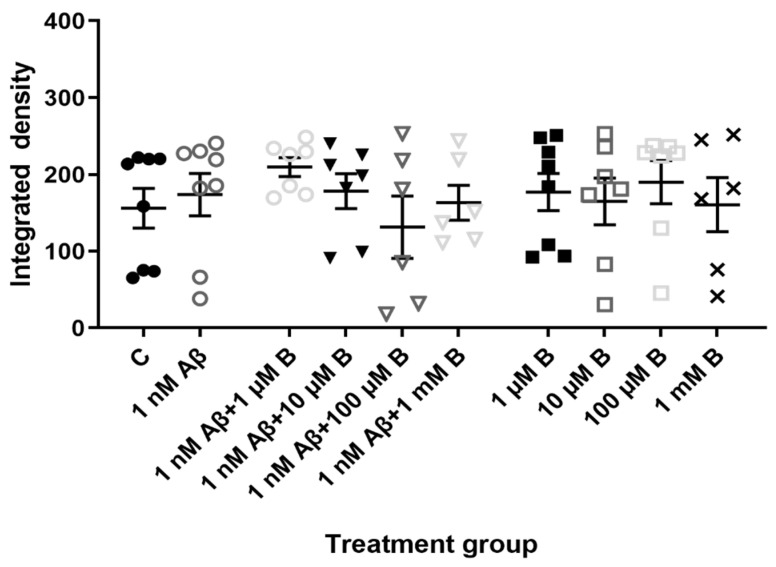
NKCC1 expression in primary mouse hippocampal neurons treated with Aβ_1-42_ and bumetanide. Expression of NKCC1 in primary hippocampal neurons at 19 DIV in vehicle controls (C) or following treatment with 1 nM Aβ_1-42_ (Aβ), 1 µM, 10 µM, 100 µM, and 1 mM bumetanide (B) with and without 1 nM Aβ_1-42_ for 5 days. Integrated density measurements from immunocytochemistry experiments were conducted using ImageJ Fiji software to quantify NKCC1 expression on primary hippocampal neurons. Data are expressed as the mean ± SEM. One-way ANOVA with Tukey’s post hoc, *n* = 6–8 for each experimental group.

Treatment with Aβ_1-42_ did not significantly change NKCC1 expression on hippocampal neurons compared to controls (Figure 4; *p* > 0.05, *n* = 8 for controls, *n* = 8 for Aβ_1-42_). In addition, there was no change in NKCC1 expression following administration of Aβ_1-42_ and bumetanide at 1 µM (*p* > 0.05, *n* = 7), 10 µM (*p* > 0.05, *n* = 7), 100 µM (*p* > 0.05, *n* = 6), or 1 mM bumetanide (*p* > 0.05, *n* = 6) compared to controls. Finally, NKCC1 expression levels were unaffected by bumetanide alone at 1 µM (*p* > 0.05, *n* = 8), 10 µM (*p* > 0.05, *n* = 7), 100 µM (*p* > 0.05, *n* = 7), and 1 mM (*p* > 0.05, *n* = 6). Overall, there was no change in NKCC1 expression across all treatment groups.

### 2.4. Effect of Aβ_1-42_ and Bumetanide on Cell Viability in Hippocampal Cultures

**Figure 5:**
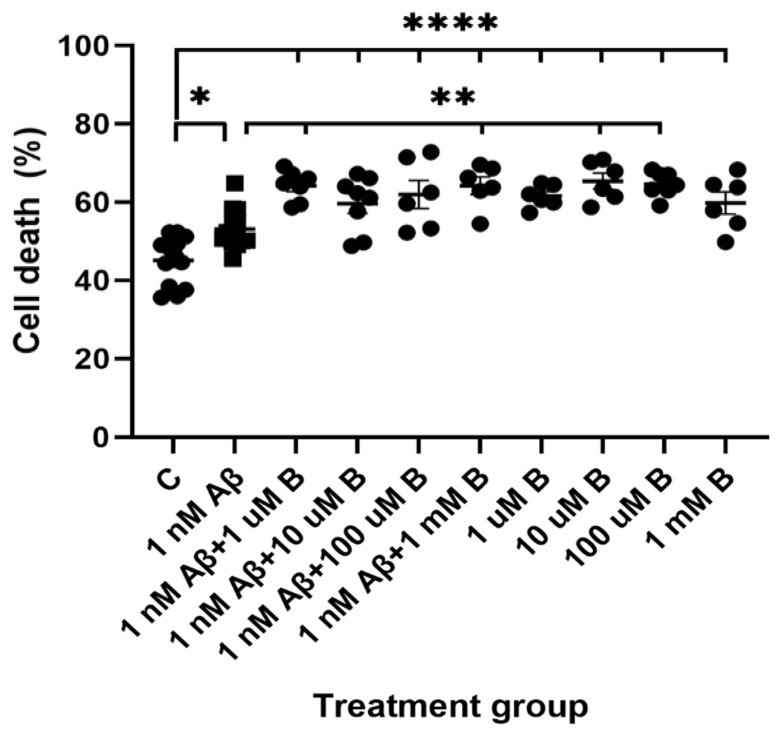
Cell viability of primary mouse hippocampal neurons following Aβ_1-42_ and bumetanide treatment. Cell death at 19 DIV in primary mouse hippocampal neurons in untreated controls (C) or following treatment with 1 nM Aβ_1-42_ (Aβ) and 1 µM, 10 µM, 100 µM, and 1 mM bumetanide (B) for 5 days. Quantification of ReadyProbes Live/Dead assay showing the percentage of cell death for each treatment group. Data is expressed as the mean ± SEM. * *p* < 0.05, **** *p* < 0.0001 compared to control, ** *p* < 0.01 compared to 1 nM Aβ_1-42_. One-way ANOVA with Tukey’s post hoc for each treatment group. *n* = 6–13 for each experimental group.

Bumetanide was added to hippocampal cultures at 1 µM, 10 µM, 100 µM, and 1 mM to determine whether the drug could prevent Aβ_1-42_-induced cell death using the ReadyProbes Live/Dead cell viability assay. After 5 days, hippocampal cultures treated with Aβ_1-42_ and bumetanide showed no improvement in neuronal survival compared to Aβ_1-42_ alone. Furthermore, all concentrations of bumetanide showed a trend towards increasing cell death, with concentrations 1 µM and 1 mM leading to a significant increase in cell death of 11% (Figure 5; ** *p* = 0.0038, *n* = 7) and 11.1% (** *p* = 0.007, *n* = 6) compared to Aβ_1-42_ alone, respectively. Interestingly, treatment with bumetanide alone significantly increased cell death to 61.5% at 1 µM (**** *p* < 0.0001, *n* = 6), 65.4% at 10 µM (**** *p* < 0.0001, *n* = 6), 64.6% at 100 µM (**** *p* < 0.0001, *n* = 7), and 59.8% at 1 mM (**** *p* < 0.0001, *n* = 6) compared to the vehicle controls. Moreover, bumetanide appeared to be more neurotoxic than Aβ_1-42_ alone, with cell death increasing by 8.4%, 12.3% (** *p* = 0.0017), 11.4% (** *p* = 0.0023), and 6.7% with 1 µM, 10 µM, 100 µM, and 1 mM bumetanide, respectively, compared to Aβ_1-42_ alone. Overall, these results suggest bumetanide is not neuroprotective against Aβ_1-42_ neurotoxicity and on its own is neurotoxic to hippocampal neurons.

### 2.5. Effect of Aβ_1-42_ on KCC2 Expression in the Mouse Hippocampus

Expression of KCC2 and NKCC1 was further investigated in an in vivo AD model by labeling mouse hippocampal sections from naïve, ACSF-injected, and Aβ_1-42_-injected mice for KCC2 and NKCC1. We determined whether the bilateral stereotaxic injection of Aβ_1-42_ affected KCC2 and NKCC1 expression in the stratum (str.) oriens, str. pyramidale, and str. radiatum of the CA1 and CA3 regions; and the str. moleculare, str. granulosum, and hilus of the DG in the mouse hippocampus compared to naïve and ACSF controls 30-days post-Aβ_1-42_-injection.

**Figure 6:**
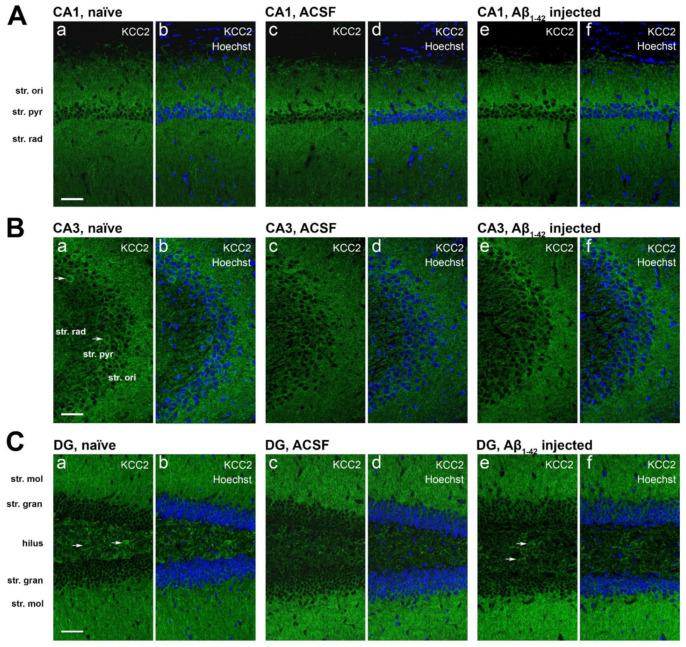
KCC2 expression in the mouse hippocampus. Confocal images of mouse hippocampal sections showing immunofluorescence labeling of KCC2 (green) and Hoechst 33342 (blue) in the mouse hippocampal regions (**A**) CA1, (**B**) CA3, and (**C**) DG of naïve (**a**,**b**), ACSF (**c**,**d**), and Aβ_1-42_-injected mice (**e**,**f**). Arrows indicate KCC2 is expressed in the soma of neurons in the CA1, CA3, and DG. Scale bar = 50 µm.

KCC2 immunoreactivity in the CA1 and CA3 regions appeared punctate in the str. oriens and str. radiatum and localized to the neuronal soma in the str. pyramidale and str. radiatum (Figure 6A,B). Neuronal cell bodies expressing KCC2 also seem to label GABAergic neurons, based on cell morphology. In the DG, KCC2 expression appears to be localized along neuronal processes in the dorsal and ventral str. moleculare, and in the soma of neurons in the dorsal and ventral str. granulosum and hilus. KCC2 immunoreactivity in the hilus showed higher density than the dorsal and ventral str. moleculare, with predominantly punctate staining (Figure 6C).

**Figure 7:**
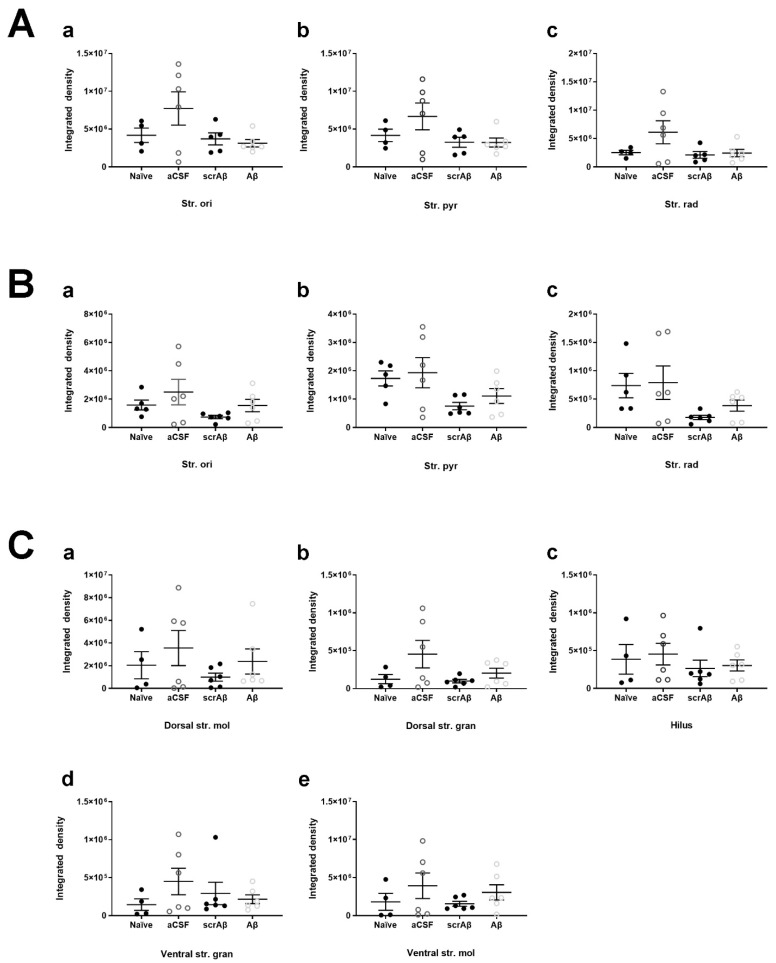
Quantification of KCC2 density in the mouse hippocampus. KCC2 density in the str. oriens (str. ori), str. pyramidale (str. pyr) and str. radiatum (str. rad) of the (**A**) CA1 and (**B**) CA3 regions, and the dorsal and ventral str. moleculare (str. mol), hilus, and dorsal and ventral str. granulosum (str. gran) of the (**C**) DG. Data is expressed as the mean ± SEM. One-way ANOVA with Tukey’s multiple comparisons test, *n* = 5–6 for each experimental group. Naïve = untreated mice; aCSF = ACSF-injected; Aβ = Aβ_1-42_-injected mice; scrAβ = scrambled Aβ_1-42_-injected mice.

**Figure 8:**
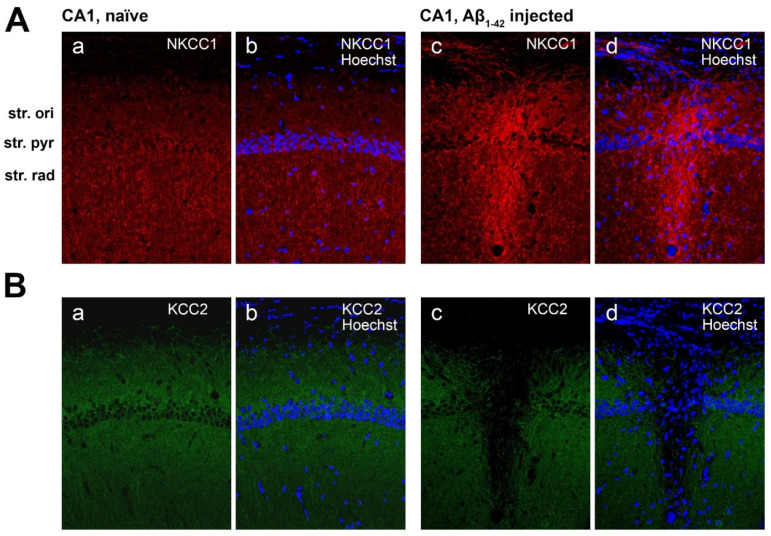
NKCC1 and KCC2 expression in the CA1 region of naïve and Aβ_1-42_-treated mice. (**A**) NKCC1 (red) and Hoechst (blue) staining in naïve mice (**a**,**b**) and Aβ_1-42_-injected mice, showing the injection site (**c**,**d**). (**B**) KCC2 (green) and Hoechst (blue) staining in naïve (**a**,**b**) and Aβ_1-42_-injected mice, showing the injection site (**c**,**d**). Scale bar = 50 µm.

KCC2 staining density did not change in the CA1 region of Aβ_1-42_-injected mice compared to naïve and ACSF controls (Figure 7A). Notably, Aβ_1-42_ appears to induce a localized downregulation of KCC2 at the CA1 injection site (Figure 8(Bc,Bd)).

In the CA3 region, KCC2 staining was unchanged in the str. oriens, str. pyramidale, and str. radiatum between naïve, ACSF, scrambled Aβ_1-42_ (scrAβ), and Aβ_1-42_-injected mice (Figure 7B). Similarly, we report no changes in KCC2 immunoreactivity in any layers of the DG between naïve, ACSF, scrAβ, or Aβ_1-42_-injected mice (Figure 7C).

### 2.6. Effect of Aβ_1-42_ on NKCC1 Expression in the Mouse Hippocampus

**Figure 9:**
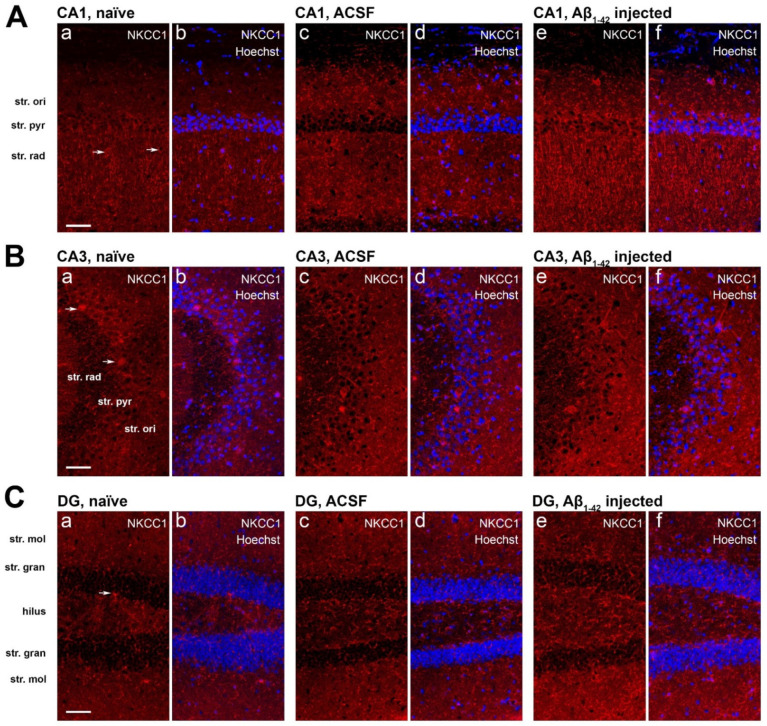
NKCC1 expression in the mouse hippocampus. Confocal images of mouse hippocampal sections showing the immunofluorescence of NKCC1 (red) and Hoechst 33,342 (blue) in the (**A**) CA1, (**B**) CA3, and (**C**) DG of naïve (**a**,**b**), ACSF (**c**,**d**), and Aβ_1-42_-injected mice (**e**,**f**). Arrows indicate NKCC1 is expressed on neuronal cell bodies in the CA1, CA3, and DG. Scale bar = 50 µm.

In the CA1 region, NKCC1 immunoreactivity appeared punctate and localized to the neuronal soma in the str. oriens and str. radiatum, along neuronal processes in the str. radiatum, and sparse on neuronal cell bodies in the str. pyramidale of naïve and ACSF mice. The punctate staining exhibited by both KCC2 and NKCC1 in the neuropil are likely to be GABAergic and glutamatergic terminals (Figure 9A). The CA3 region exhibited similar immunoreactivity but with greater somatic staining in the str. pyramidale (Figure 9B). NKCC1 staining in the DG is also punctate in the dorsal and ventral str. moleculare and hilus, and weakly somatic in the dorsal and ventral str. granulosum, suggesting localization to GABAergic neurons based on neuronal morphology (Figure 9C).

**Figure 10:**
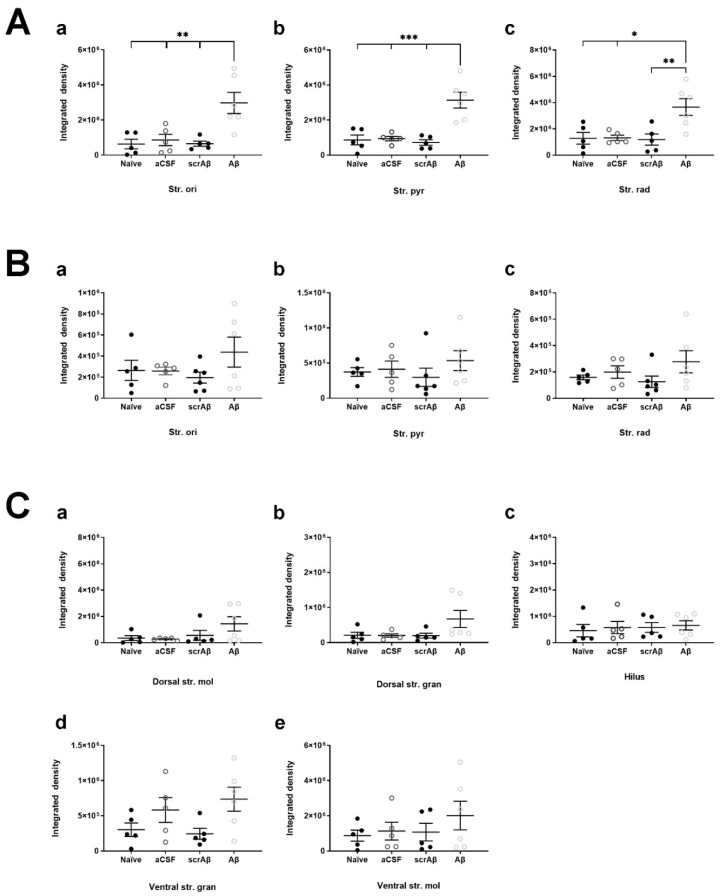
Quantification of NKCC1 density in the mouse hippocampus. (**A**) NKCC1 density in the str. oriens (str. ori), str. pyramidale (str. pyr) and str. radiatum (str. rad) of the (**A**) CA1 and (**B**) CA3 regions, and the dorsal and ventral str. moleculare (str. mol), hilus, and dorsal and ventral str. granulosum (str. gran) of the (**C**) DG. Data is expressed as the mean ± SEM. One-way ANOVA with Tukey’s multiple comparisons test, *n* = 4–6 for each experimental group (* *p* ≤ 0.05; ** *p* ≤ 0.01; *** *p* ≤ 0.001). Naïve = untreated mice; aCSF = ACSF-injected; Aβ = Aβ_1-42_-injected mice; scrAβ = scrambled Aβ_1-42_-injected mice.

In the CA1 region of the Aβ_1-42_-injected mice NKCC1 immunoreactivity significantly increased in the str. oriens compared to naïve controls (** *p* = 0.0034, *n* = 5), ACSF (** *p* = 0.0082, *n* = 5), and scrAβ-injected mice (** *p* = 0.0037, *n* = 5). In the str. pyramidale and str. radiatum NKCC1 expression levels were greater in Aβ_1-42_-injected mice compared to naïve (*** *p* = 0.0003 and * *p* = 0.0117, *n* = 5), ACSF (*** *p* = 0.0004 and * *p* = 0.0128, *n* = 5), and scrAβ (*** *p* = 0.0002 and ** *p* = 0.0085, *n* = 5) groups 30-days post- Aβ_1-42_-injection, respectively (Figure 10A). As for KCC2, the effect of Aβ_1-42_ on NKCC1 expression is localized to the CA1 injection site but more extended as NKCC1 expression appears to increase specifically around the needle track (Figure 8(Ac,Ad)).

There was no significant change in NKCC1 expression density in any of the hippocampal layers in the CA3 region (Figure 9B and Figure 10B) and DG (Figure 9C and Figure 10C) of Aβ_1-42_-injected mice compared to naïve and ACSF controls.

## 3. Discussion

The present study investigated the expression of the CCCs, KCC2 and NKCC1 in primary mouse hippocampal neurons and mouse hippocampal tissue, and the effect of bumetanide, an NKCC1 inhibitor, on KCC2 and NKCC1 expression in an in vitro AD mouse model. We also investigated whether bumetanide was neuroprotective against Aβ_1-42_ toxicity. This study showed KCC2 and NKCC1 expression is unaffected by Aβ_1-42_ and bumetanide in vitro in mouse primary neuronal cultures, but in vivo NKCC1 is upregulated in the CA1 region of the hippocampus. Finally, cell survival in an in vitro AD model is not improved with bumetanide treatment.

We demonstrated that KCC2 is present on both immature and mature hippocampal neurons, represented by 7 and 19 DIV cultures respectively, predominantly on the cell soma and along neuronal processes. Indeed, we expected to observe KCC2 expression in our cultures as KCC2 function becomes apparent at 13–14 days in hippocampal culture. At this point there is a gradual increase in dendritic E_GABA_ currents, indicating neuronal maturation and a switch in GABAergic neurotransmission from excitatory to inhibitory [39]. Quantitative mRNA studies in rat brains also show hippocampal pyramidal neurons highly express KCC2, thus our findings are consistent with previous studies [29]. Williams and colleagues report a similar expression pattern in rat cerebellar Purkinje neurons, as KCC2 was observed in the neuronal soma, plasma membranes, and dendrites [31]. Hippocampal cultures from embryonic day 17 mice also express KCC2 as early as 3 DIV in the soma of immature neurons, which spreads to the neurites by 15 DIV [40]. Notably, dendritic KCC2 expression levels were not apparent at 3 DIV in a study by Ludwig et al. [40], but we observed expression on neuronal processes at 7 DIV. The KCC2 detected on neuronal processes in our immature cultures could be a non-functional form as it has been suggested cultured neurons lack the required activation factor present in vivo [39]. The expression on neuronal processes coincides however, with the development of functional GABAergic synapses in 7–14 DIV cultures [41]. To verify the functionality of KCC2 expressed in our immature cultures it would be informative conducting an electrophysiology study to detect changes in E_GABA_ in conjunction with dendritic KCC2 expression. Nonetheless, our findings confirm the neuron-specific nature of KCC2 through the colocalization with NeuN.

Furthermore, we report GABAergic and glutamatergic neurons express KCC2 at 7 DIV, particularly on the cell body and proximal neuronal processes. KCC2 expression on cholinergic neurons could not be reported as cholinergic neurons were not detected in our cultures. The ChAT antibody used in this study has been previously used with success, suggesting our cultures were void of cholinergic neurons [42]. The hippocampus receives most of its cholinergic innervation from the medial septal nucleus and the diagonal band of Broca but is not void of cholinergic neurons itself [43]. Sparse, non-pyramidal, cholinergic neurons have been reported in the rat hippocampal formation with excitatory synapses [44]. Interestingly, studies indicate the septo-hippocampal pathway is not predominantly cholinergic. In addition, the presence of choline acetyltransferase in the rat hippocampal formation suggests the hippocampal formation receives intrinsic cholinergic innervation [43,45]. Therefore, we can postulate that our cultures did not contain cholinergic neurons.

Immunofluorescent imaging of the mouse hippocampus showed KCC2 expression is punctate in the str. oriens and str. radiatum; and localized to the neuronal soma in the str. pyramidale and str. radiatum in the CA1 and CA3 regions. Meanwhile, KCC2 is largely present on neuronal processes and soma in the DG. The punctate staining of KCC2 in the neuropil likely represent GABAergic and glutamatergic terminals. High expression of KCC2 on the dendrites of PV-interneurons in the str. oriens and str. alveus of the CA1 and CA3 regions, and in the molecular layer of the DG has been previously reported in the rat hippocampus [32]. It has also been suggested that KCC2 is localized to excitatory synapses in pyramidal cells of the hippocampus [32].

In the in vitro AD mouse model, we conclude that Aβ_1-42_ does not influence KCC2 expression since the relative level of KCC2 was unchanged following Aβ_1-42_ administration. Bumetanide also had no effect on KCC2 expression. Other studies show KCC2 expression is often unaffected in pathophysiological conditions while NKCC1 is upregulated [21]. The duration of Aβ_1-42_ and bumetanide treatment in our cultures may have not been sufficient to induce a change in KCC2 expression since cultures could not be prolonged further to investigate this. Several studies suggest KCC2 expression is regulated by transcriptional factors, as trophic factors such as brain-derived neurotrophic factor (BDNF) or insulin-like growth factor 1 are involved in KCC2 developmental upregulation [27,40,46]. In hippocampal slices KCC2 mRNA is downregulated in CA1 pyramidal neurons by a transcriptional mechanism involving BDNF and tyrosine receptor kinase B (TrkB) signaling [27]. Investigating the effect of BDNF on KCC2 expression in this model would be difficult considering the wide spectrum of effects growth factors have on neuronal maturation. Analysis of mouse hippocampal tissue in an in vivo AD mouse model confirmed KCC2 expression is unaffected in the CA1, CA3, and DG regions of the mouse hippocampus 30-days post-Aβ_1-42_ injection. Most interestingly, it appeared as though KCC2 was downregulated specifically at the CA1 injection site. Full length APP is capable of modulating KCC2 expression by stabilizing KCC2 on the cell membrane and thus reducing its degradation, and by post-transcriptional mechanisms [47,48]. Perhaps once cleaved into Aβ_1-42_ this may switch into more localized modulation of KCC2 expression. KCC2 might become less stable on the cell membrane and more prone to degradation following Aβ_1-42_-injection [47]. KCC2 may also be susceptible to transcriptional downregulation as it has a high turnover rate once present on the plasma membrane [27]. Lack of expressional change outside the injection site may be due to the aforementioned reason that KCC2 is normally transcriptionally regulated [27,40,46]. Future endeavors could focus on investigating the prolonged effect of bumetanide on hippocampal KCC2 expression following Aβ_1-42_ administration in the in vivo model, to observe whether bumetanide can affect the apparent localized modulation of KCC2. However, the possible neurotoxic effects of the drug have to be explored as well.

We also report NKCC1 is expressed on neuronal cell bodies and processes in immature and mature neurons, with mature neurons predominantly exhibiting expression on the cell body. NKCC1 also appeared to be expressed on non-neuronal cells, which we speculate are endothelial cells in our cultures. As NKCC1 is shown to be expressed by a diverse population of cells such as vascular endothelial cells, this finding was not unexpected [28,35,49]. Immature neurons highly express NKCC1 to promote Cl^−^ influx, a less negative E_GABA_, and depolarizing GABA activity. Throughout development, NKCC1 expression diminishes as GABA becomes hyperpolarizing, but is still present [18]. Thus, we expected to observe NKCC1 in both immature and mature neurons with more expression at 7 DIV [49]. We also report both GABAergic and glutamatergic neurons express NKCC1. NKCC1 has been implicated in the maturation of excitatory and inhibitory synapses in CA1 pyramidal cells as development of glutamatergic and GABAergic synapses were delayed in NKCC1 knock-out mice [50]. Plotkin et al. [49] also reported weak NKCC1 expression in the cell bodies and processes of GAD65-positive Purkinje cells in the cerebellum, which increased by postnatal day 14.

In the mouse hippocampus NKCC1 was expressed along neuronal processes of pyramidal neurons; and punctate in the str. oriens and str. radiatum of the CA1 region. Previous studies in the adult rat brain have also shown specific expression of NKCC1 on the dendrites of CA1 and CA3 pyramidal neurons [37,49]. The punctate staining present in the neuropil, as for KCC2, likely represent GABAergic and glutamatergic terminals in the CA1, CA3, and DG of the hippocampus. Meanwhile, in neuronal bodies of the str. pyramidale of the CA1 and CA3 sub-regions NKCC1 expression is sparse. It has been previously reported that NKCC1 expression is high on individual neurons early in postnatal development but demonstrates localized expression on the apical dendrites of mature pyramidal neurons [49]. Similarly, another study demonstrated NKCC1 mRNA expression remains moderate in the pyramidal cell layer of the CA1 and CA3 regions of the rat hippocampus even beyond postnatal day 15 [51]. This current study begins to fill the gap in the literature regarding KCC2 and NKCC1 expression in different neuronal populations, but more research is needed to further characterize their expression in excitatory and inhibitory neurons.

In the in vitro AD mouse model, there was no change in NKCC1 expression following a 5-day exposure to Aβ_1-42_, suggesting Aβ_1-42_ does not affect NKCC1 expression. The addition of bumetanide at four concentrations had no effect on NKCC1 expression either alone or in conjunction with Aβ_1-42_ treatment. It is plausible that NKCC1 is regulated by transcriptional mechanisms like KCC2. NKCC1 activation relies on phosphorylation and thus kinase activity [52]. A study found proline–alanine-rich STE20-related kinase, or PASK, is the kinase responsible for regulating NKCC1 expression [53]. And like KCC2, BDNF has been shown to downregulate NKCC1 expression in the hippocampus of rat status epilepticus models, likely through transcriptional mechanisms [54]. As for KCC2, treatment duration may have not been sufficient to induce any expressional changes in NKCC1. One of the limiting factors of primary cell culture is the inability to prolong cultures. As our cultures could not be maintained beyond 4 weeks, it was not possible to extend the treatment duration. Treatment could also not be delayed as it is vital that a sufficient number of viable cells remain to conduct reliable and meaningful analyses on these cultures. Neurons begin to become less viable in older cultures meaning it was most optimal to begin treatment at 14 DIV and end treatment at 19 DIV. In vivo experiments are less limited by time constraints in the way they can be maintained for longer periods and may be a better representative of AD progression. Thus, it is important to examine hippocampal NKCC1 expression after a longer exposure time to Aβ_1-42_, particularly in an in vivo model. In the in vivo AD mouse model NKCC1 expression appeared to undergo layer and region-specific increases in the CA1 region, particularly in the CA1 injection site of Aβ_1-42_-injected mice 30-days post-injection. Specifically, NKCC1 density was enhanced in the str. pyramidale and str. radiatum of the CA1 region of Aβ_1-42_-injected mice compared to naïve and ACSF controls. The inhibitory-to-excitatory switch in GABAergic transmission may be a local effect of Aβ_1-42_ and the limited duration of cell culture experiments may be insufficient for Aβ_1-42_ to have an effect, which supports the speculation that NKCC1 is transcriptionally regulated like KCC2. Elucidating which kinases are involved in NKCC1 upregulation will be challenging as several kinases have been implicated in regulating NKCC1 expression [53]. Regarding the functional consequences we postulate that altered NKCC1 expression contributes to the inhibitory-to-excitatory switch in GABAergic transmission in AD, thus exacerbating an E/I imbalance. Seizure models have demonstrated NKCC1 promotes excitation as bumetanide attenuated epileptiform activity in in vitro hippocampal slices, and electrographic seizures in neonatal rats in vivo [33]. Western blot analysis and whole-cell patch-clamp recordings in R6/2 and YAC128 HD mice have also shown increased NKCC1 expression, reduced KCC2 expression, and a depolarized E_GABA_ in mature CA1 hippocampal neurons, which consequently promotes GABA_A_-mediated neuronal excitability [24].

Finally, we showed that bumetanide is not neuroprotective against Aβ_1-42_-induced cell death in this in vitro AD model and may even exacerbate cell death. We hypothesized that the E/I imbalance and depolarizing GABA activity in AD may be influenced by altered expression of KCC2 and NKCC1. Increased NKCC1 expression would increase the intracellular Cl^−^ concentration, disrupt inhibition, and increase cellular excitation [33]. The neuroprotective effects of bumetanide was observed in both in vitro and in vivo models of cerebral ischemia [55,56]. The drug produced a 25% decrease in infarct volume following middle cerebral artery occlusion [56] by possibly preventing significant intracellular Cl^−^ rises during reoxygenation and/or reducing of Na^+^ influx via NKCC1 and preventing excitotoxicity. Therefore, in AD bumetanide might also protect against Aβ_1-42_-induced excitotoxicity by restoring the E/I imbalance. DS mouse models have shown bumetanide could rescue hippocampal-dependent memory and synaptic plasticity [21]. In addition, Dargaei et al. [24] has shown NKCC1 is increased while KCC2 is decreased in the hippocampus of HD mouse models, which also exhibit a more depolarized E_GABA_, loss of inhibitory drive, and increased neuronal excitability. As treatment with bumetanide abolished these deficits, it became apparent that NKCC1 triggered GABA_A_-mediated excitation [24]. However, in the current study primary hippocampal cultures treated with bumetanide had greater cell death than those treated with Aβ_1-42_ alone. This suggests bumetanide is capable of inducing cell death, and the presence of NKCC1 in mature neurons, although lowly expressed, are necessary for normal physiological function. Therefore, blocking its activity via bumetanide may be detrimental and promote cell death. Indeed, impairing NKCC1 function has been shown to disrupt K^+^ influx and secretion into the endolymph involved in hearing in NKCC1 knock-out mice, leading to deafness and balance impairments [57]. Presently, NKCC1 inhibition via bumetanide may prompt an ionic imbalance by abolishing NKCC1-mediated Cl^−^ influx, promoting KCC2-mediated Cl^−^ efflux and thus GABA_A_R-mediated hyperpolarization. Increased inhibition in a DS mouse model has been associated with hippocampal learning and memory deficits [58]. As cell death was considerably higher following bumetanide administration, the drug may not be suitable as a potential therapy in AD or diseases involving altered KCC2 or NKCC1 expression, as regions with normal KCC2 and NKCC1 expression may be affected. This was unexpected as in R6/2-HD mice 10 µM of bumetanide, representing the second lowest concentration used in our study, administered via micro-osmotic infusion pumps restored GABAergic hyperpolarization and improved hippocampal learning and memory deficits [24]. Another study by Schliess et al. [59] used 5 µM of bumetanide which had no effect on platelet-derived growth factor (PDGF)-induced proliferation in rat hepatic stellate cells (HSCs) at 14 DIV. However, in the perfused rat liver bumetanide inhibited the volume-regulatory net K^+^ uptake leading to cell shrinkage, which was postulated to promote generation of reactive oxygen intermediates and oxidative stress [59]. Although this did not negatively affect the viability of HSCs, it may be detrimental to neuronal survival. It is also possible the bumetanide-induced neurotoxicity observed in our study is related to non-specific toxicity. Pond et al. [55] reported higher concentrations of bumetanide abolished its neuroprotective ability following oxygen–glucose deprivation, leading to non-specific toxic effects. The authors suggested that while 10 µM bumetanide was sufficient to selectively inhibit NKCC1, the maximally effective concentration of 100 µM, that protected CA1 neurons, may also lead to KCC2 inhibition [55,60]. Such consequences were confirmed when addition of a KCC2 inhibitor exacerbated cell death, swelling, and damage to hippocampal slices [55]. In our study, 1 µM bumetanide was sufficient to induce cell death, suggesting another mechanism may be involved in neurotoxicity.

In summary, we demonstrate that KCC2 and NKCC1 expression is not altered by Aβ_1-42_ and bumetanide does not protect primary hippocampal neurons from Aβ_1-42_ induced neurotoxicity. Importantly, bumetanide reduces cell viability in the presence of Aβ_1-42_ and is highly neurotoxic on its own to hippocampal primary neurons. NKCC1 is upregulated in the CA1 region of the hippocampus following Aβ_1-42_ administration in an in vivo AD mouse model. These findings indicate more research is required to elucidate the effects of Aβ_1-42_ on KCC2 and NKCC1 expression in the human AD brain to explore if targeting CCCs might offer a therapeutic approach for AD, as we demonstrate altered CCC expression may be a contributing factor to the E/I imbalance in AD. If bumetanide has a therapeutic potential for AD, the neurotoxicity on hippocampal neurons suggest caution as to its suitability, therefore more work is required to understand its mechanism of action on neurons.

## 4. Materials and Methods

### 4.1. Animals

All experiments were approved by the University of Auckland Animal Ethics Committee (AEC; Approval number 006155 and 001586). Mice were housed at the University of Auckland Vernon Jansen Unit under standard laboratory conditions and maintained in a 12 h light-dark cycle with food and water *ad libitum*. Postnatal (P0) male C57/BL/6 mice were used to culture mouse hippocampal neurons while male C57BL/6 mice at 18-months of age were used in vivo experiments (RRID:IMSR_JAX:000664; The Jackson Laboratory, Bar Harbor, ME, US). A total of 75 mice were used form the in vitro experiments and 24 aged mice used for the in vivo study. The aged mice were categorized into four groups, NC (*n* = 6), ACSF- injected (*n* = 6), scrAβ_1–42_-injected (*n* = 6) and Aβ_1–42_-injected (*n* = 6).

### 4.2. Primary Hippocampal Cell Culture

Cell culture procedures were adapted from Beaudoin et al. [61] with adjustments outlined by Vinnakota et al. [62]. P0 C57/BL/6 male mouse pups were euthanized by decapitation and had the hippocampus dissected, removing the surrounding meninges in the process. The hippocampi were then washed in Hank’s balanced salt solution (HBSS; Invitrogen 14175095, New York, NY, USA) containing 1 mM sodium pyruvate, 10 mM HEPES buffer, and 0.1% glucose. Tissue was dissociated using 0.25% trypsin (Invitrogen) and Basal Medium Eagle (Invitrogen, 21010046, New York, NY, USA), and 0.04% DNAse. A fire-polished glass pipette was used to dissociate the cells and produce a homogenous cell suspension by trituration prior to plating in 24-well plates (Falcon, 353047, Corning, NY, USA) coated with poly-D-lysine (Sigma, P9155, Saint Louis, MO, USA). Cells were placed in a 37 °C, 5%CO_2_/95% O_2_ cell culture incubator for 4 h to allow for cell adhesion. After this, the plating media was removed and replaced with fresh Neurobasal medium (Invitrogen, 21103049, New York, NY, USA), 2 µg/mL gentamicin (Invitrogen, 15710072), and 2 mM glutamine (Invitrogen, 25030081, Paisley, Scotland, UK). Every 3-4 days half the media was exchanged. To inhibit glial cell proliferation and reduce the presence of astrocytes in culture, 5 µM of cytosine arabinofuranoside (araC; Sigma, 251010, Darmstadt, Germany) was used at 3 days in vitro (DIV). Neurons could be maintained for up to 4 weeks, sufficient to differentiate, and develop axons, dendrites, and synaptic connections.

### 4.3. Aβ_1-42_ Preparation

Aβ_1-42_ was produced as a recombinant protein with maltose binding protein (MBP) to establish a highly soluble recombinant fusion protein, which was then expressed in *Escherichia coli*. The product was then purified on an amylose column which binds to the MBP portion. The pure fusion protein was extracted from the amylose resin using maltose and concentrated using ammonium sulphate precipitation. Factor X protease was used to cleave the carrier MBP off the fusion protein, then the Aβ_1-42_ protein was further isolated and purified using hydrophobic chromatography with 0–50% *v*/*v* acetonitrile/0.1% *v*/*v* TFA using FPLC. Fractions with pure Aβ_1-42_ were detected immunologically using antibodies against residues 17–24 of Aβ_1-42_, which were lyophilized to remove the solvent. The expected molecular ion for the product was determined using mass spectrometry. Prior to in vitro application, Aβ_1-42_ was dissolved in artificial cerebrospinal fluid (aCSF), diluted culture medium, and aged by incubating in a 37 °C water bath for 48 h to increase the neurotoxicity of Aβ_1-42_ by facilitating the formation of toxic soluble aggregates [42,63]. Whereas prior to in vivo application and stereotaxic injection, Aβ_1-42_ was dissolved in aCSF and ‘aged’ as above. The optimal incubation time for Aβ_1-42_ preparations to produce toxic oligomers varies between preparations but ranges from 48–120 h. Yeung et al. [64] previously performed SDS-PAGE experiments to confirm the ‘aging’ and aggregate formation of Aβ_1-42_. Western blots of an aging profile of Aβ_1-42_ showed the Aβ_1-42_ monomer decreases and the oligomer appears by 48 h in non-dissociating gels. Whereas in SDS gels, the dimer and trimer of Aβ_1-42_, and a higher molecular weight oligomer, can be observed at 5 days. This preparation of Aβ_1-42_ has been shown to be highly neurotoxic both in vitro and in vivo [42,62,63,64,65,66].

### 4.4. Drug Treatments

Primary mouse hippocampal neurons were treated with 1 nM of aged Aβ_1-42_ at 14 DIV, when neurons represent an ‘adult’ state, for 5 days to establish an in vitro AD mouse model [60]. Cultured neurons at 14 DIV also express GABA signalling components characteristic for a mature adult state [62]. The NKCC1 inhibitor, bumetanide (Sigma, B3023, Saint Louis, MO, USA), was diluted in culture medium to make a 1 mM stock solution and dissolved by alternating between sonicating for 10 min at 60 °C and heating in a water bath at 60 °C 4 times. Cell viability was determined following treatment with 1 nM Aβ_1-42_ and 1 µM, 10 µM, 100 µM, and 1 mM bumetanide in 500 µL of culture medium for 5 days.

### 4.5. Measuring Cell Viability Using the ReadyProbes Cell Viability Imaging Kit

Cell viability was determined 5 days after treatment with Aβ_1-42_ and/or bumetanide using the ReadyProbes Cell Viability Kit (ThermoFisher, R37609, Eugene, OR, USA), as per the manufacturer’s instructions. Wells were randomly allocated for treatment with different bumetanide concentrations (1 µM, 10 µM, 100 µM, and 1 mM) alone or with 1 nM Aβ_1-42_, 1 nM Aβ_1-42_ alone, or as controls. Control wells were treated with an equal volume of culture medium, which was used to dissolve Aβ_1-42_ and bumetanide, to mimic treatment administration. At least 8-13 wells were allocated per treatment group. At 19 DIV, 15 µL of the NucBlue^®^ Live reagent and 15 µL of the NucGreen^®^ Dead reagent was added to each well containing 500 µL of culture medium. Cells were returned to the incubator for 15 min to facilitate reagent staining prior to imaging. The NucBlue^®^ reagent labels the nuclei of all cells (live and dead) to fluoresce blue, while the NucGreen^®^ reagent labels the nuclei of dead cells only to fluoresce green. Cells were imaged using the ImageXpress Micro XLS (Version 5.3.0.5, Molecular Devices, San Jose, CA, USA) using the DAPI filter to acquire the NucBlue^®^ staining and the FITC filter from the Quad5 filter cube to acquire the NucGreen^®^ staining. Each well was imaged across 16 regions of interest using the 20×/0.45 NA CFI Plan Fluor ELWD ADM (phase contrast) objective lens. Cells were counted manually and based on neuronal morphology to determine the total number of cells based on NucBlue^®^ staining and the total number of dead cells based on NucGreen^®^ staining.

### 4.6. Fluorescent Immunocytochemistry

**Figure 11:**
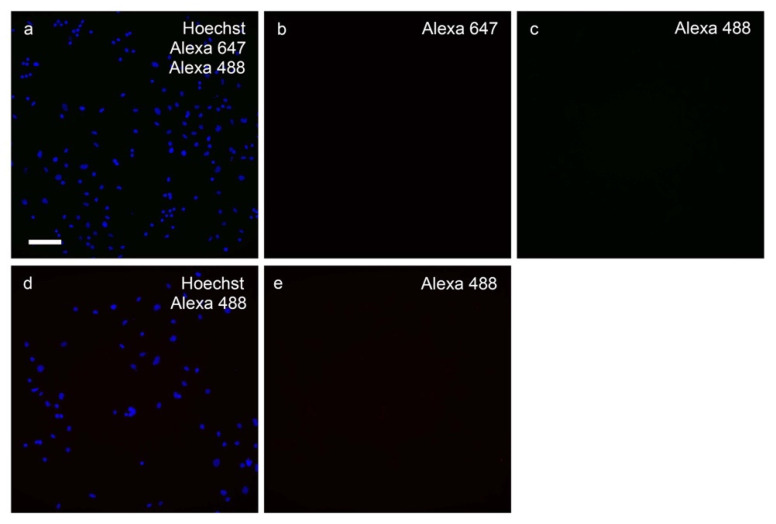
The omission of the primary antibodies resulted in complete absence of the immunoreactivity. Primary hippocampal neuronal cultures were labeled with (**a**) Hoechst 33,342 (blue); (**b**) goat anti-mouse Alexa 647 (red); (**c**) goat anti-guinea pig Alexa 488 (green); (**d**) Hoechst 33,342 (blue); and (**e**) goat anti-rabbit Alexa 488 (green). Scale bar = 100 µm.

Fluorescent immunocytochemistry was performed on hippocampal cultures once fixed at the end of the treatment phase using 4% paraformaldehyde (PFA) and stored in PBS-azide at 4 °C. KCC2 and NKCC1 expression levels were characterized in control cultures, and cultures treated with Aβ_1-42_ and/or bumetanide. Cells were also stained for glutamatergic neurons using vesicular glutamate transporter 1 (VGluT1), GABAergic neurons using glutamic acid decarboxylase (GAD)65/67, and cholinergic neurons using choline acetyltransferase (ChAT). Cells were washed thrice for 5 min in PBS containing 0.05% Tween 20 (PBT) and incubated in PBS containing 0.2% Triton-X 100 (PBST) for 5 min on a slow rocker at room temperature (RT). Cells were then washed thrice for 5 min with PBT and blocked with 1% BSA-PBT (immunobuffer) for 1 h at RT before adding primary antibodies for KCC2 (mouse monoclonal, NeuroMab, 75-013; 1:2000), NKCC1 (mouse monoclonal, DSHB, T4-C 2ea; 1:5000), NeuN (rabbit monoclonal, Millipore, ABN78; 1:1000), VGluT1 (guinea-pig monoclonal, Millipore, ABN5905; 1:1000), GAD65/67 (rabbit monoclonal, Sigma, G5163; 1:1000), and ChAT (goat monoclonal, Chemicon, AB144P; 1:2000). Specificity of the primary antibodies has been tested and reported previously (KCC2, [47,67,68,69,70,71]; NKCC1, [69,70,72,73,74]; GAD65/67, [75,76]; ChAT, [77]). The mouse monoclonal, DSHB, T4-C 2ea developed by Lytle, C./Forbush III, B. was obtained from the Developmental Studies Hybridoma Bank, created by the NICHD of the NIH and maintained at The University of Iowa, Department of Biology, Iowa City, IA 52242. Primary antibodies were diluted in immunobuffer and added for 24 h at 4 °C on a slow rocker. The next day, cells were washed thrice for 5 min with PBT at RT and incubated in Alexa Fluor secondary antibodies diluted 1:500 in immunobuffer for 1 h at RT on a slow rocker. The secondary antibodies used were goat anti-mouse 647 (Invitrogen, A21236), goat anti-rabbit 488 (Invitrogen, A11034), goat anti-guinea-pig 488 (Invitrogen, A11073), donkey anti-mouse 647 (Invitrogen, A31571), and donkey anti-goat 488 (A11055). Then cells were washed with PBT for 10 min, incubated with Hoechst33342 (Molecular Probes, H3570; 1:10,000) for 5 min at RT on a slow rocker, then washed with PBT for 5 min and twice with PBS for 5 min at RT. Control wells were incubated in immunobuffer in place of primary antibody to determine the degree of non-specific secondary antibody binding. Wells with the primary antibody omitted were run in tandem with each experiment. The omission of the primary antibodies resulted in complete absence of immunoreactivity (Figure 11).

The cells were stored in PBS and imaged using the ImageXpress Micro XLS (Version 5.3.0.5, Molecular Devices, San Jose, CA, USA) across nine regions of interest per well with the 20×/0.45 NA CFI Plan Fluor ELWD ADM (phase contrast) objective lens. The DAPI, TxRed, and CY5 excitation filters were used to acquire Hoechst, NeuN, and KCC2 or NKCC1 staining, respectively. Image acquisition settings were optimized for each antibody and identical across all experimental and treatment replicates.

Signal intensity was measured over the whole image across nine regions of interest per well specifically on neurons. For each image, a mask was created based on NeuN staining to identify neurons only. The NeuN mask was then overlaid onto the corresponding KCC2 or NKCC1 fluorescent images and density measurements captured based on the NeuN mask to determine neuronal transporter expression only. The raw intensity data was normalized by dividing it by the area as the area for density measurements differed between sites. Densitometry measurements of KCC2 and NKCC1 staining, based on a NeuN staining mask was conducted using ImageJ Fiji software (U.S. National Institutes of Health, Bethesda, MD, USA). It was ensured the researchers were blinded to the treatment groups during analysis and the threshold was kept identical across all the wells analysed. Three experimental replicates, each with 8–13 wells per treatment group, were used for this experiment. Two hippocampi dissected from one mouse was sufficient to culture 12 wells of primary neurons in a 24-well plate.

### 4.7. Mouse In Vivo Experiments

The surgery and treatments, collection, and preparation of mouse tissue used in this study was done as described in Calvo-Flores Guzmán et al. [65]. Mice were anaesthetized by injection with 75 mg/kg of ketamine and 1 mg/kg of domitor prior to surgery. Stereotaxic surgery was performed to bilaterally inject 1 µL of 20 µM of ‘aged’ Aβ_1-42_, scrambled Aβ_1-42_ (scrAβ; AS-25382, AnaSpec, Fremont, CA, USA), or ACSF in to the CA1 region of the hippocampus, located relative to the bregma (anterior-posterior, −2.0 mm; medial-lateral, ±1.3 mm; dorsal-ventral, −2.2 mm), at a speed of 0.5 µL/min [42,65]. Anaesthesia was abolished post-surgery by subcutaneously administering 1 mg/kg of antisedan. Thirty days post-Aβ_1-42_ administration animals were anaesthetized via ketamine and domitor (12.5 mL/kg) and transcardially perfused with 4% PFA. Brains were then extracted, postfixed in 4% PFA solution for 2 h at RT and then incubated in 30% sucrose in TBS (pH 7.6; 0.05 M Tris-HCl, 0.15 M NaCl) overnight at 4 °C, cut into 30 μm sections using a freezing microtome, and stored in antifreeze solution at −20 °C. Mice were treated with aCSF, Aβ_1-42_ alone, or scrAβ. aCSF and no treatment (naïve) mice were used as controls [65].

### 4.8. Fluorescent Immunohistochemistry

**Figure 12:**
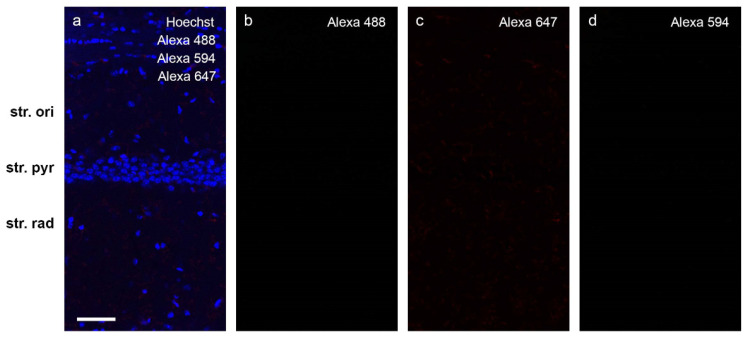
The omission of the primary antibodies resulted in complete absence of the immunoreactivity. Mouse hippocampal tissue was labeled with (**a**) Hoechst 33,342 (blue); (**b**) goat anti-rabbit Alexa 488 (green); (**c**) goat anti-mouse Alexa 647 (red); and (**d**) goat anti-guinea pig Alexa 594 (yellow). Scale bar = 50 µm.

Fluorescent free-floating immunohistochemistry was performed on mouse hippocampal brain sections. KCC2 and NKCC1 expression levels were characterized in naïve control mice, ACSF-injected control, Aβ_1-42_-injected, and scrAβ-injected mice. Two coronal mouse brain sections containing the hippocampus were washed once for 5 min in PBS and twice for 5 min in PBS containing 0.1% Triton X-100 (PBST) at RT. Sections were blocked for 1 h at RT using PBS containing 10% goat serum and 0.1% Triton X-100 prior to adding primary antibodies for KCC2 (rabbit monoclonal, Abcam, AB97502; 1:500), NKCC1 (mouse monoclonal, DSHB, T4-C 2ea; 1:1000), and NeuN (guinea-pig monoclonal, Millipore, ABN90P; 1:1000). Primary antibodies were diluted in 1% BSA in PBST and added for 24 h at 4 °C. The following day sections were washed thrice for 10 min with PBS and incubated in Alexa Fluor secondary antibodies diluted 1:500 in PBST for 2 h at RT on a slow rocker. The secondary antibodies used were goat anti-mouse 647 (Invitrogen, A21236), goat anti-rabbit 488 (Invitrogen, A11034), and goat anti-guinea pig 594 (Invitrogen, A11076). Sections were then incubated in Hoechst33342 (Molecular Probes, H3570; 1:10,000) for 30 min at RT and washed thrice for 10 min in PBS. Sections were mounted on gelatin-coated slides, allowed to air-dry overnight, rehydrated using Milli-Q water, coverslipped using Mowiol488 mounting medium (Calbiochem, 475904) and finally sealed with nail varnish. Control sections were incubated in 1% BSA in PBST in place of primary antibody to determine the degree of non-specific secondary antibody binding. The omission of the primary antibodies resulted in complete absence of immunoreactivity (Figure 12).

Both hippocampi on each section was imaged using the Zeiss 710 confocal laser-scanning microscope (Carl Zeiss, Jena, Germany) at the CA1, CA3, and DG regions at 20× magnification using the dry 20×/0.8NA objective lens (Carl Zeiss, Jena, Germany), and using the 405 nm laser for Hoechst, 488 nm laser for AlexaFluor^®^488, 561 nm laser for AlexaFluor^®^594, and the 633 nm laser for AlexaFluor^®^647. Hippocampal regions and layers were identified based on Hoechst staining and relative location. Images were obtained directly at, and to the left and right of the stereotaxic injection site in Aβ_1-42_, ACSF, and scrAβ injected mice in the CA1 region. Integrated density measurements and analysis was conducted using the ImageJ Fiji software (U.S. National Institutes of Health, Bethesda, MD, USA) in the following areas in each layer: 21,352 μm^2^ for the CA1 region, 4761 μm^2^ for the CA3 region, and 12,295 μm^2^ for the DG to quantify KCC2 and NKCC1 expression. Intensity measurements were performed in the following CA1 and CA3 layers: str. oriens, str. pyramidale, and str. radiatum; and in the following DG layers: molecular layer, granular layer, and hilus. Intensity was measured adjacent to the injection site, 50–500 µm from the needle track [64]. After background subtraction and greyscale threshold determination, density measurements were performed for each marker from all region of interests. The experimenter was blinded to the experimental groupings to eliminate any bias during the experiment, including during image acquisition and analysis.

### 4.9. Statistical Analysis

For cell viability, immunocyto- and histochemistry experiments, a one-way ANOVA with a Tukey’s multiple comparisons test was used to determine differences between treatment groups. All statistical analyses were conducted using GraphPad Prism version 8.2.1 (GraphPad Prism Software, San Diego, CA, USA) with a *p* ≤ 0.05 considered significant. All figures and images were prepared using Adobe Photoshop CC (2021) (Adobe Systems Software, San Jose, CA, USA). All experimental data is presented as the mean ± the Standard Error of Mean (SEM).

## Data Availability

The original contributions presented in the study are included in the article, further inquiries can be directed to the corresponding author.

## Sample Availability

Not available.
